# Supplementary figures and images for: Features extracted using tensor decomposition reflect the biological features of the temporal patterns of human blood multimodal metabolome
Source: PLoS One. 2023 Feb 15;18(2):e0281594. doi: 10.1371/journal.pone.0281594 (PMC9931158; doi:10.1371/journal.pone.0281594)

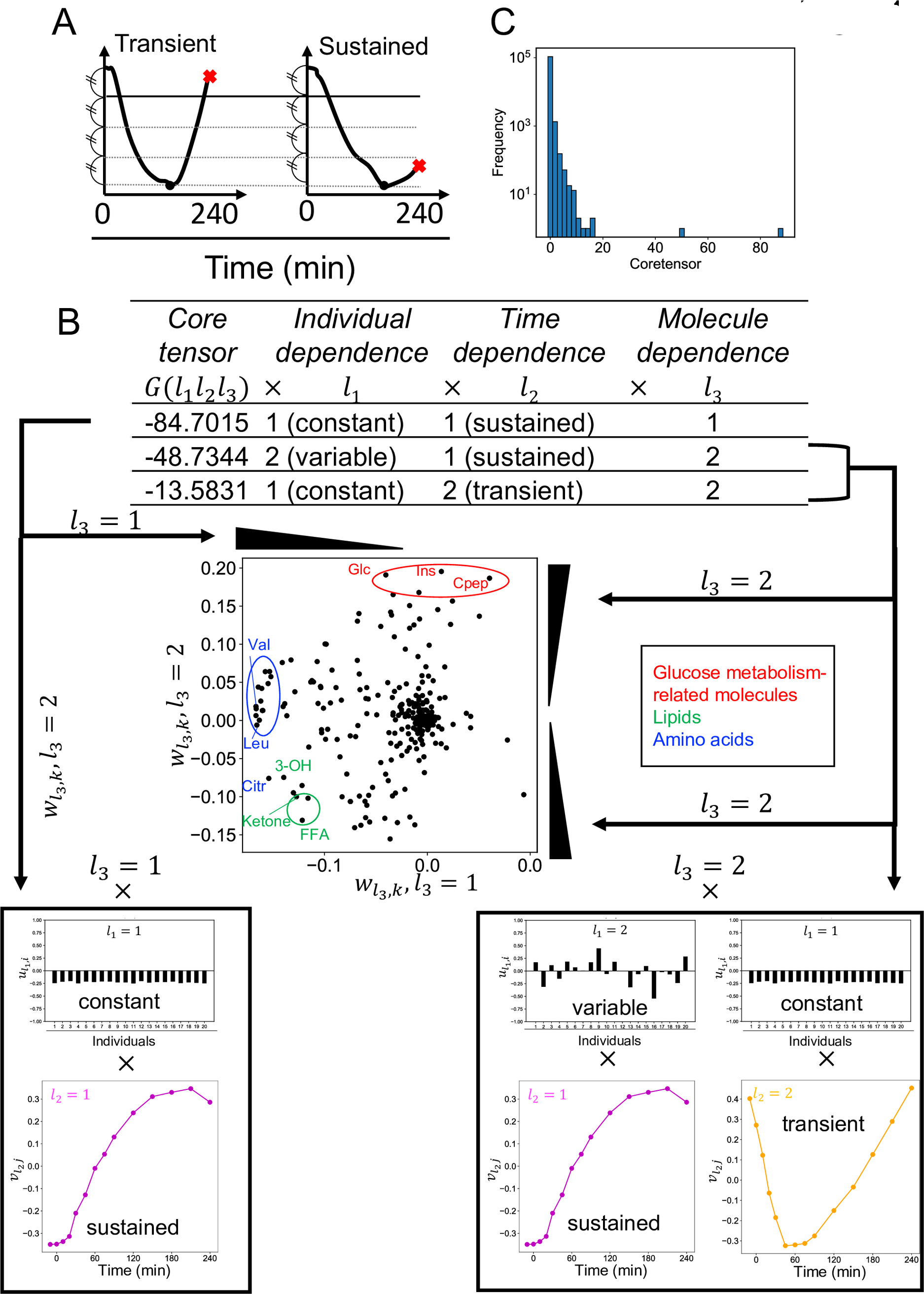

Supplement: S1 Fig — A The definition of temporal pattern B Procedures for selecting a set of molecules with specific time-dependent or individual-dependent properties C Distribution of absolute values of the core tensor. (TIF) [file pone.0281594.s001.tif]

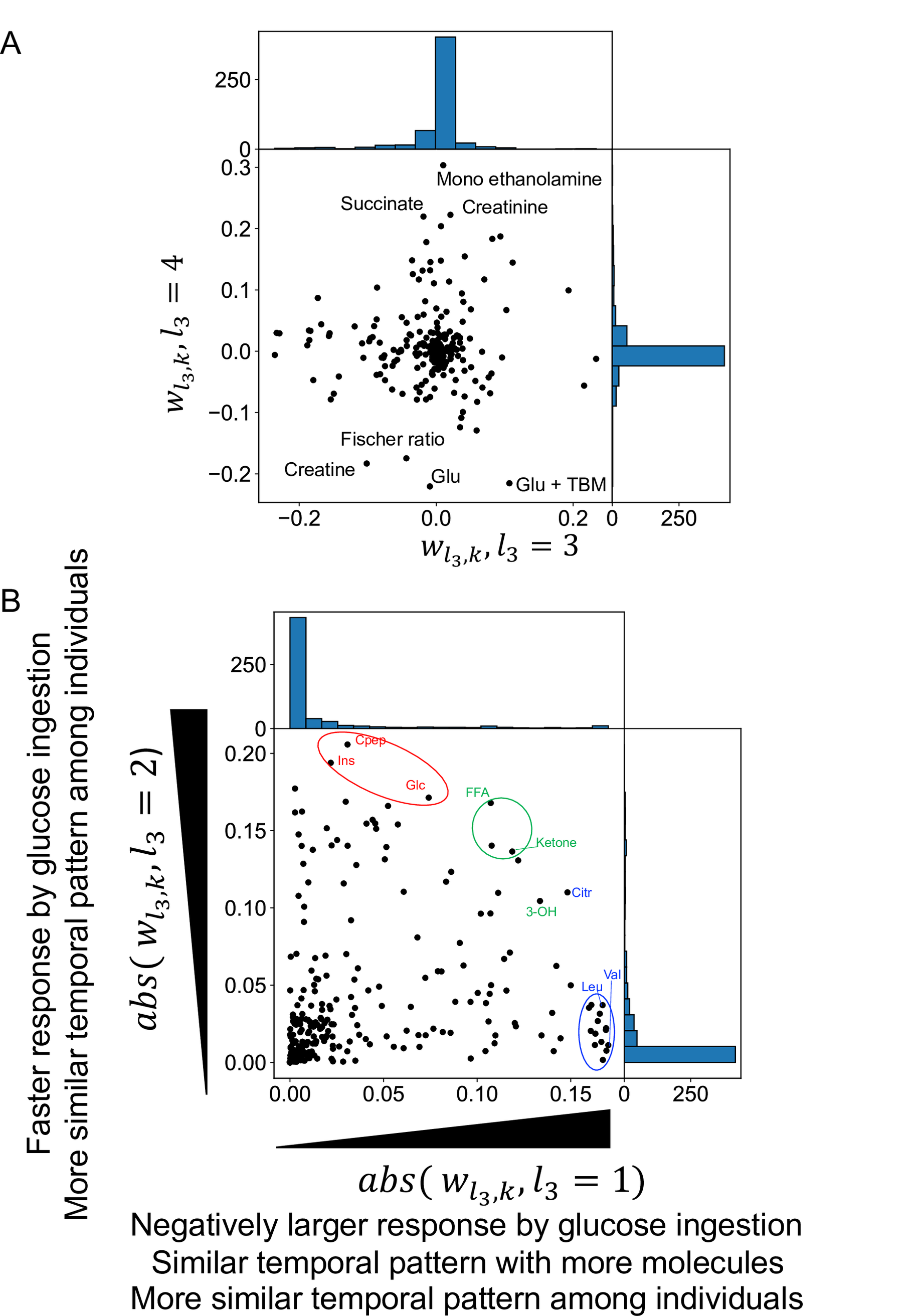

Supplement: S2 Fig — A The distribution of the molecule-related singular vectors (wl3k,l3=1,2). B The distribution of the absolute value of the molecule-related singular vectors (wl3k,l3=1,2). Representative molecules are labeled. Abbreviations for the representative molecules are as follows: Cit, citrulline; CRP, C-reactive peptide; FFA, free fatty acid; 3-OH, 3-hydroxybutyric acid; Ketone, Total ketone body; Glc, glucose; Ins, Glu, glutamic acid; insulin; Leu, leucine; Val, valine. The label colours correspond to the metabolic group list. (TIF) [file pone.0281594.s002.tif]

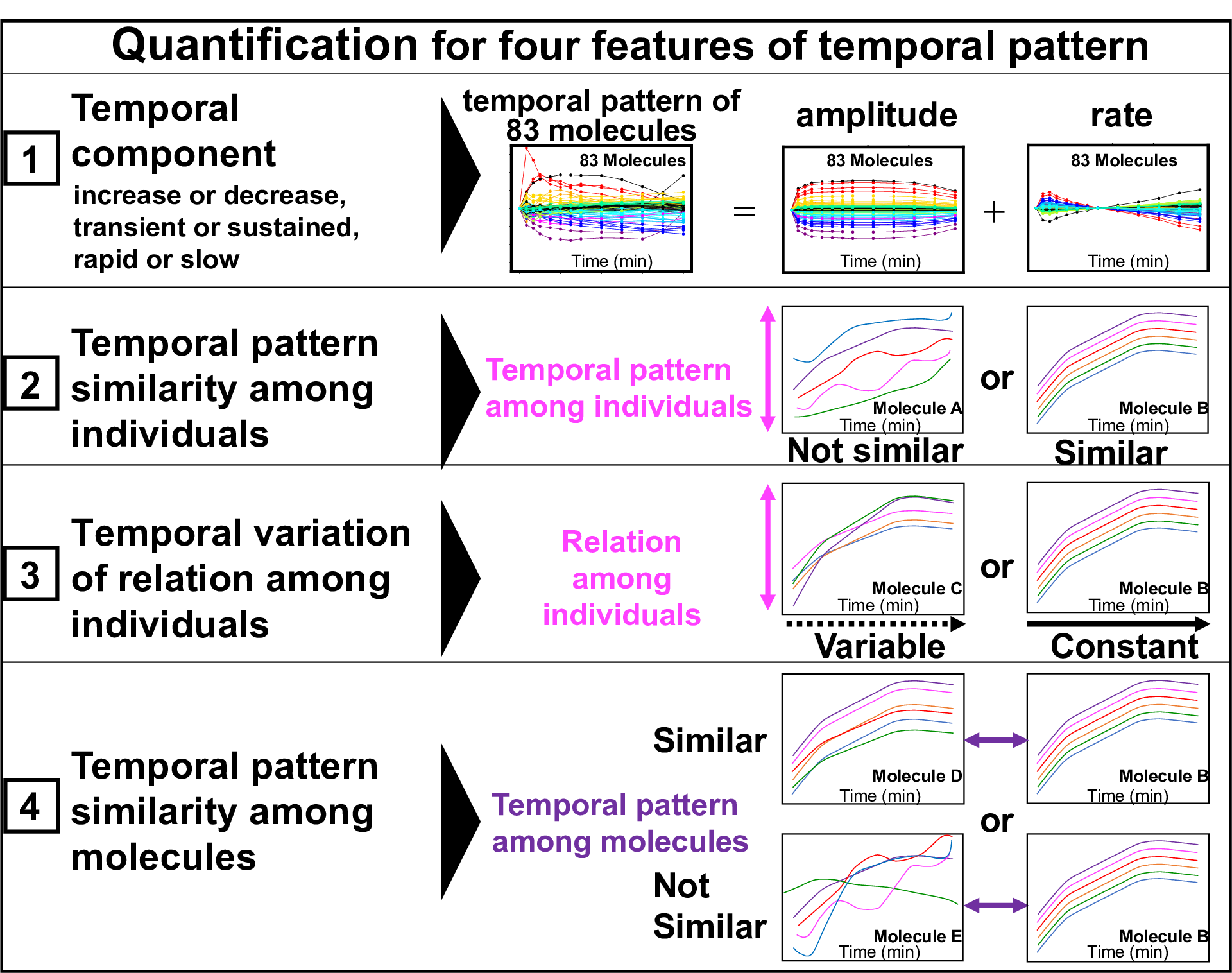

Supplement: S3 Fig — In our earlier study, we used hypothesis-driven analysis and characterized the temporal patterns among individuals and among molecules by the hypothesis-driven analysis with four features [4]: the decomposability into “amplitude” and “rate” components, the similarity of temporal patterns among individuals, the relationship among individuals’ over time, and the similarity of temporal patterns among molecules. (TIF) [file pone.0281594.s003.tif]

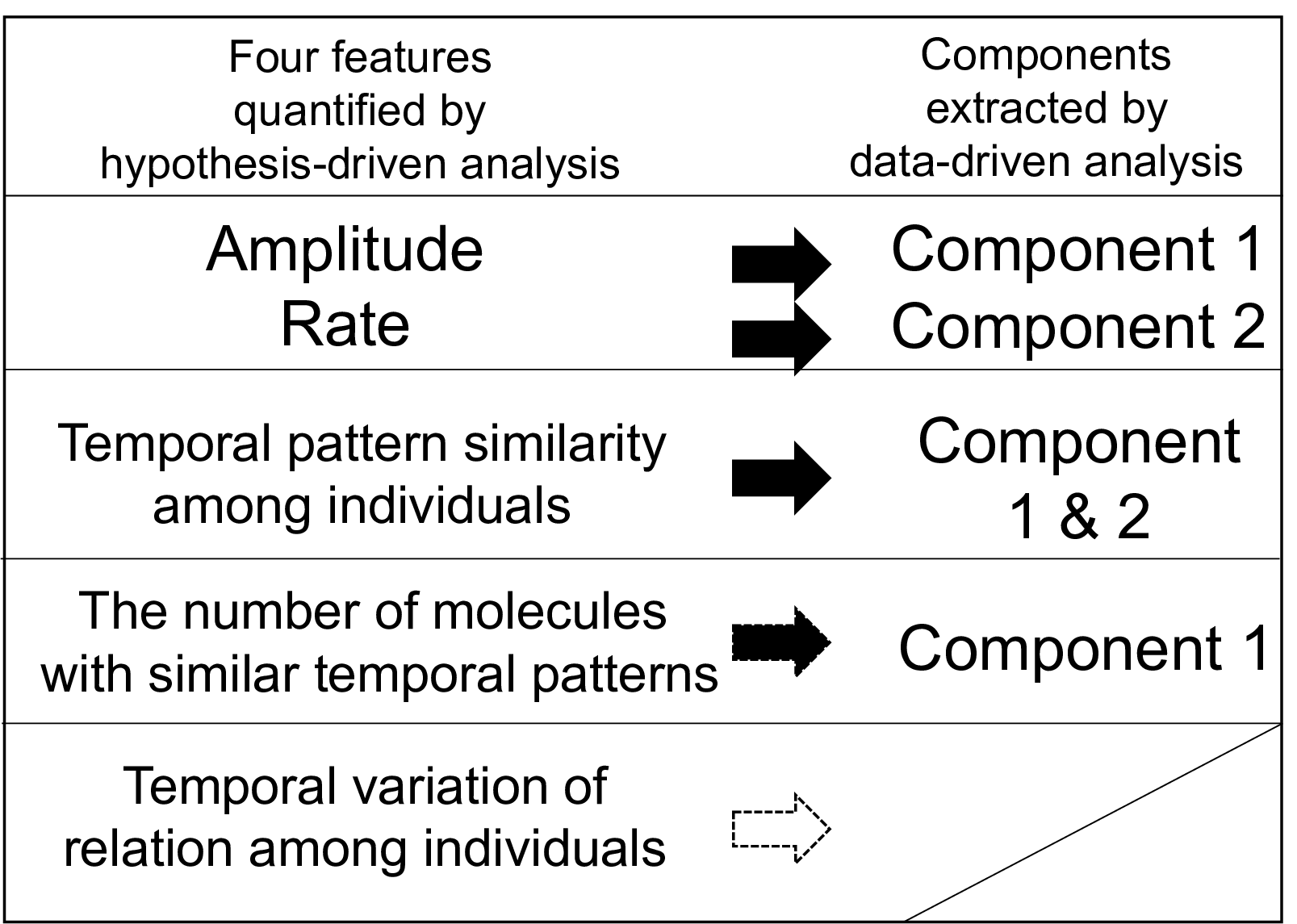

Supplement: S4 Fig — (TIF) [file pone.0281594.s004.tif]

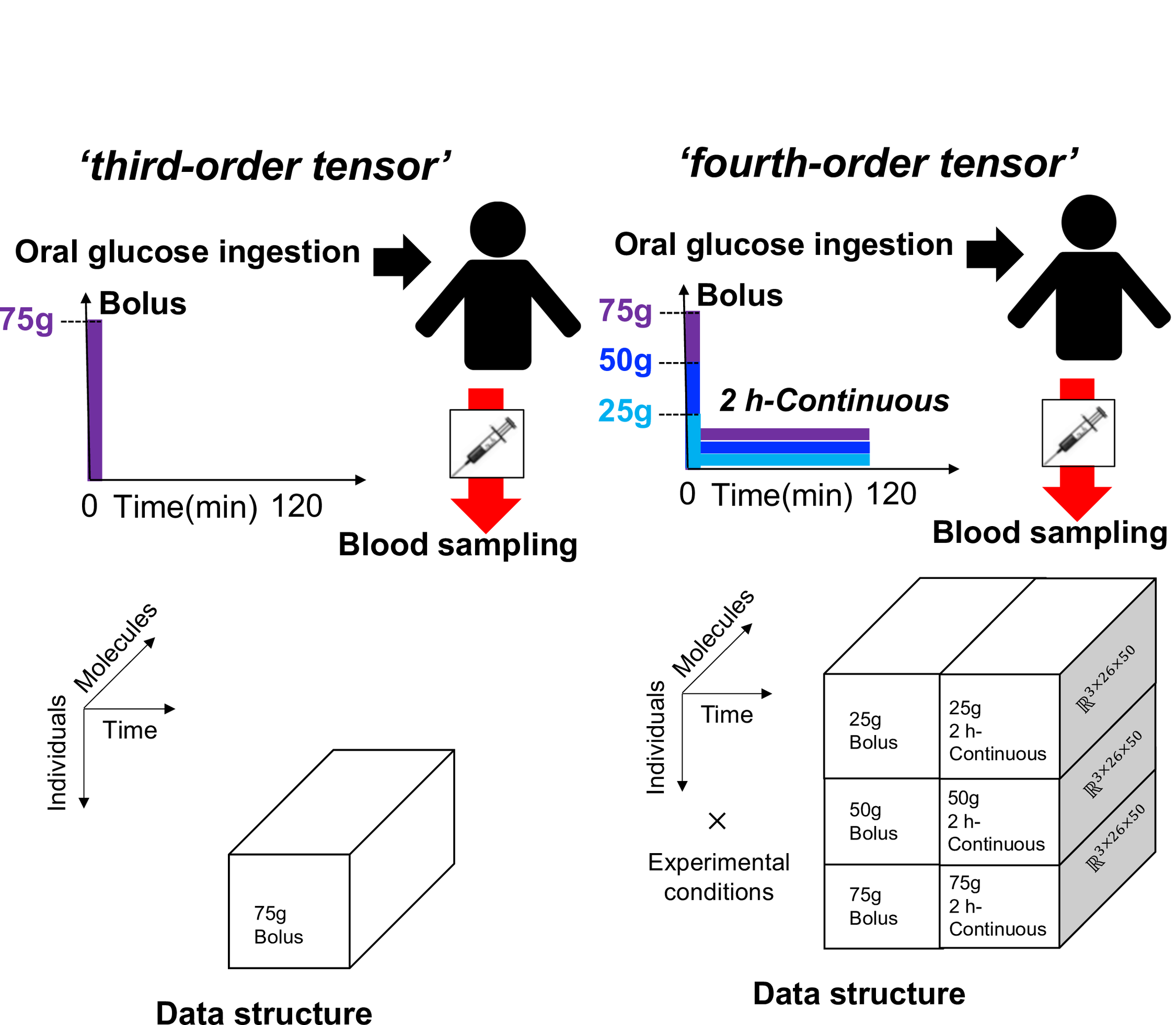

Supplement: S5 Fig — For ‘third-order tensor’, 20 susbjects orally ingested 75g glucose in bolus ingestion. The data structure has three axes: individual × time × molecule. The data represent the concentration changes at 26 time points 10, 20, 30, 45, 60, 75, 90, 120, 150, 180, 210, 240 min before and after ingestion as previously described [4]. For ‘fourth-order tensor’, Three subjects orally ingested glucose with three doses 75, 50, and 25 g in two durations of bolus and 2 h continuous ingestion. The data structure has four axes: individual × time × experimental condition × molecule. The data represent the concentration changes at 26 time points (-5, 0, 10, 20, 30, 40, 50, 60, 70, 80, 90, 100, 110, 120, 130, 140, 150, 160, 170, 180, 190, 200, 210, 220, 230, 240 minutes) from 5 min before fasting to 240 min after glucose ingestion for 40 molecules in three healthy subjects, in six different experimental conditions. (TIF) [file pone.0281594.s005.tif]

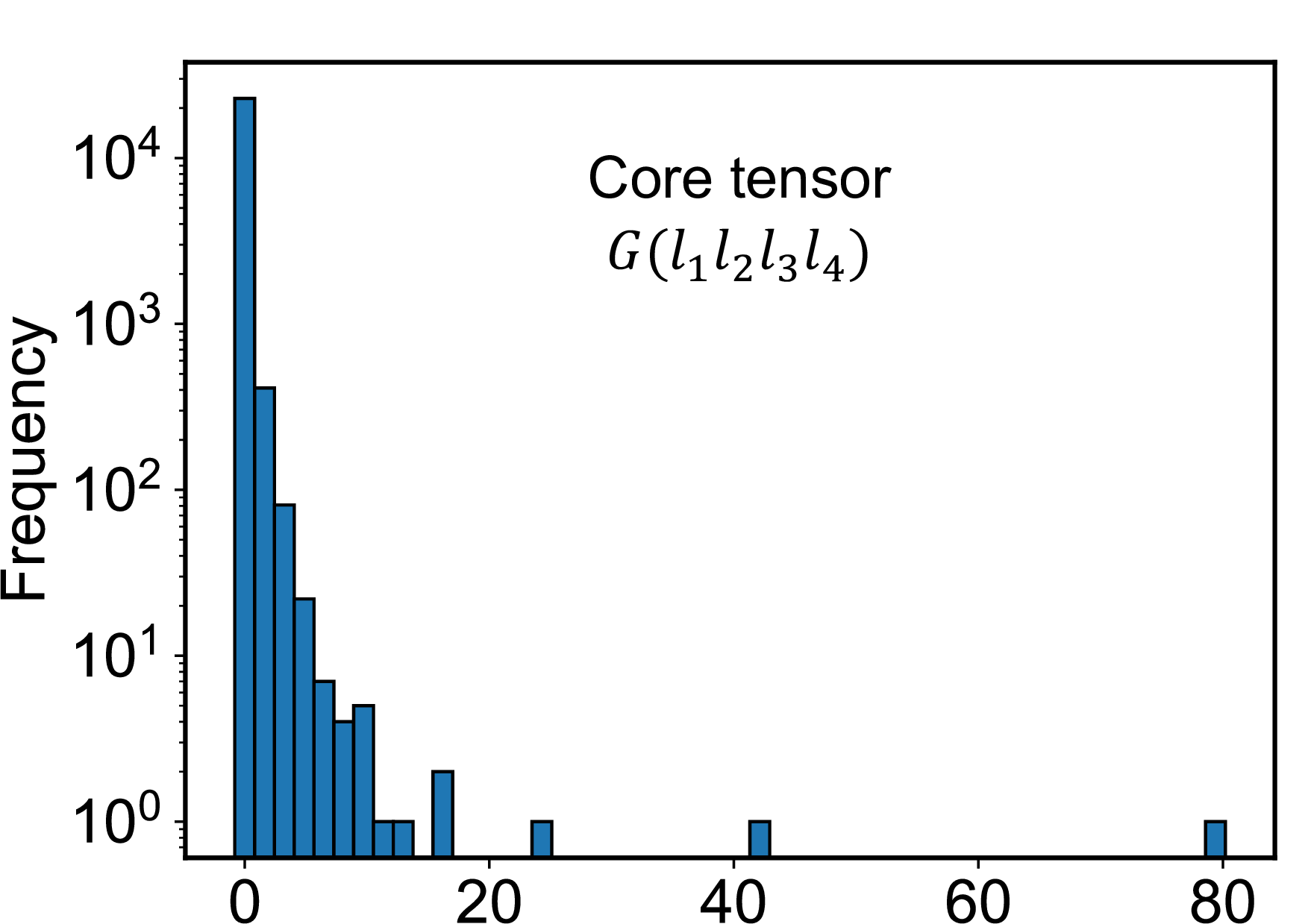

Supplement: S6 Fig — (TIF) [file pone.0281594.s006.tif]
